# Dynamics of polymers in coarse-grained nematic solvents†

**DOI:** 10.1039/d4sm00968a

**Published:** 2024-11-06

**Authors:** Zahra K. Valei, Karolina Wamsler, Alex J. Parker, Therese A. Obara, Alexander R. Klotz, Tyler N. Shendruk

**Affiliations:** a School of Physics and Astronomy, The University of Edinburgh Peter Guthrie Tait Road Edinburgh EH9 3FD UK t.shendruk@ed.ac.uk; b School of Mathematics, Loughborough University Leicestershire LE11 3TU UK; c Department of Physics and Astronomy, California State University, Long Beach Long Beach California 90840 USA

## Abstract

Polymers are a primary building block in many biomaterials, often interacting with anisotropic backgrounds. While previous studies have considered polymer dynamics within nematic solvents, rarely are the effects of anisotropic viscosity and polymer elongation differentiated. Here, we study polymers embedded in nematic liquid crystals with isotropic viscosity *via* numerical simulations to explicitly investigate the effect of nematicity on macromolecular conformation and how conformation alone can produce anisotropic dynamics. We employ a hybrid multi-particle collision dynamics and molecular dynamics technique that captures nematic orientation, thermal fluctuations and hydrodynamic interactions. The coupling of the polymer segments to the director field of the surrounding nematic elongates the polymer, producing anisotropic diffusion even in nematic solvents with isotropic viscosity. For intermediate coupling, the competition between background anisotropy and macromolecular entropy leads to hairpins – sudden kinks along the backbone of the polymer. Experiments of DNA embedded in a solution of rod-like fd viruses qualitatively support the role of hairpins in establishing characteristic conformational features that govern polymer dynamics. Hairpin diffusion along the backbone exponentially slows as coupling increases. Better understanding two-way coupling between polymers and their surroundings could allow the creation of more biomimetic composite materials.

## Abstract 2

Hairpins serve as crucial conformational degrees of freedom for polymers embedded in nematic backgrounds, affecting their diffusivity and revealing the intricate interplay between macromolecular conformation and anisotropic nature of environment.

## Graphical abstract

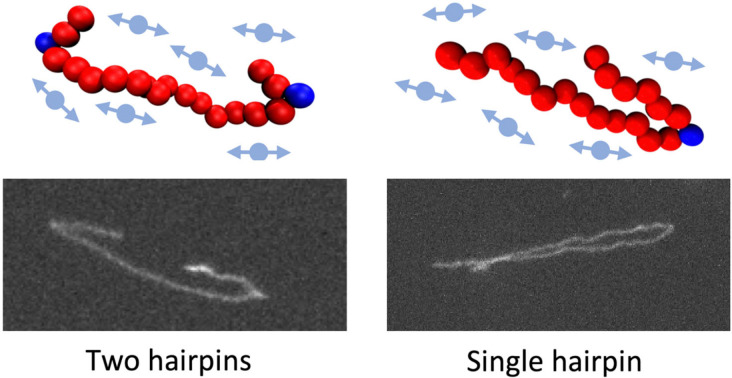

## 1 Introduction

Composite materials are ubiquitous in biology, with their versatile and functional macroscopic properties arising from greater complexity compared to single constituent counterparts.^1^ Biopolymer composites, such as microtubules in filamentous actin,^2^ filamentous bacteriophages in pathogenic biofilms,^3^ polysaccharides components in cell walls,^4^ chiral chitin^5^ and mucus,^6–8^ exemplify mesoscale constituents suspended within already complex soft material backgrounds. Of these, polymers embedded in liquid crystalline solvents (so-called hypercomplex liquid crystals^9^) are particularly interesting because they idealize the competition between the broken symmetry of the surrounding medium and the tendency of the suspended phase to maximize entropy.

Macromolecules in good, isotropic solvents possess many internal degrees of freedom, such that entropy maximization encourages them to adopt random-coil configurations on scales greater than their Kuhn length. On the other hand, polymers suspended in liquid crystalline solvents can be highly extended along the nematic director,^10^ with fluctuations away from perfect alignment quantified by the orientation distribution of the main polymer axis^11^ and by the Odijk deflection length.^12^ In fact, not only do they align with the director, but semiflexible polymers are observed to possess enhanced orientational order compared even to the background liquid crystal. This surprising result has been experimentally demonstrated by direct single-molecule visualizations of semiflexible F-actin filaments, worm-like micelles and neurofilaments suspended in nematic phase solutions of rod-like virus particles,^13^ as well as conjugated polymers in 5CB.^14^ Increasing contour length further increases the measured polymer orientational order,^13^ though increasing segmentation within conjugated polymers decreases order.^15^ Accompanying these changes in conformations are changes to the dynamics. Polymers in nematic surroundings exhibit anisotropic diffusion,^16,17^ but it is not immediately clear if the anisotropic diffusion is due to the fluid's anisotropic viscosity or if it arises because of the elongation of the polymer.

While the interactions between component molecules lead to nematic alignment and complex dynamics, they are fundamentally difficult to simulate because of the division of time scales between the nematic surroundings and the polymeric solute. Indeed for this reason, numerical studies of the dynamics of semiflexible polymers within an ensemble of many nematically ordered chains are more common than simulations of single macromolecules within mesophase nematics.^18,19^ The profound lack of numerical techniques for efficiently simulating macromolecules in liquid crystalline solvents demands novel simulation techniques be utilized if we wish to fundamentally understand the physical principles that lead to the unusual mechanical properties of binary mixtures of semiflexible polymers and low-molecular-weight nematogens.

Motivated by single-molecule microscopy images of DNA suspended in a solution of nematically ordered fd viruses, we introduce a hybrid mesoscopic simulation method based on molecular dynamics (MD) and nematic multi-particle collision dynamics (N-MPCD). This hybrid approach employs standard MD for simulating semiflexible macromolecules and coarse-grained N-MPCD for modelling the nematic fluid, including diffusion, director fluctuations and hydrodynamic interactions. We investigate the configurational dynamics of a single chain by examining the relationship between hairpins along the backbone of polymer and polymer configuration, as well as the diffusion of hairpins. We observe that the hairpins themselves diffuse along backbone of the polymer, while the polymer exhibits anisotropic diffusion even though the fluid viscosity is isotropic. Our simulations provide evidence that the macromolecular conformation can be just as significant as the anisotropy of the viscosity in governing the diffusional anisotropy ratio.

## 2 Experiments

Here, we qualitatively examine the effects of a nematic solvent on DNA molecules that are approximately 1000 persistence lengths long. We use T4 DNA (169 kbp) stained with YOYO-1 fluorescent dye, leading to a contour length of approximately 60 μm, compared to a persistence length of approximately 50 nm. The DNA is embedded in a solution of rod-like fd viruses, which are 880 nm long and 6.6 nm in diameter‡‡Nematic solutions were obtained from Christopher Ramirez and Zvonimir Dogic at the University of California, Santa Barbara.. The virus solution concentration is 20 μg mL^−1^ in an aqueous solvent with an ionic strength of 20 mM, placing them in the weakly cholesteric nematic phase.^20^ DNA is stained in the same ionic conditions and mixed with the virus solution at a 4 : 1 ratio, leaving the viruses in a nematic phase despite the lower concentration. Nematicity is verified by viewing the solution under cross-polarized microscopy; it is birefringent but not iridescent, consistent with previous characterization of the cholesteric phase.^20^

Because nematic fd virus solutions are a poor solvent for DNA,^13^ the equilibrium configuration of the DNA molecules is a diffraction limited globule. However when shear is applied, the molecules are transiently elongated within the nematic. Molecules are sheared by confining a solution of fd-DNA between a glass slide and an unsealed glass cover slip, separated by approximately 50 μm, and sliding the cover slip with respect to the slide.

**Fig. 1:**
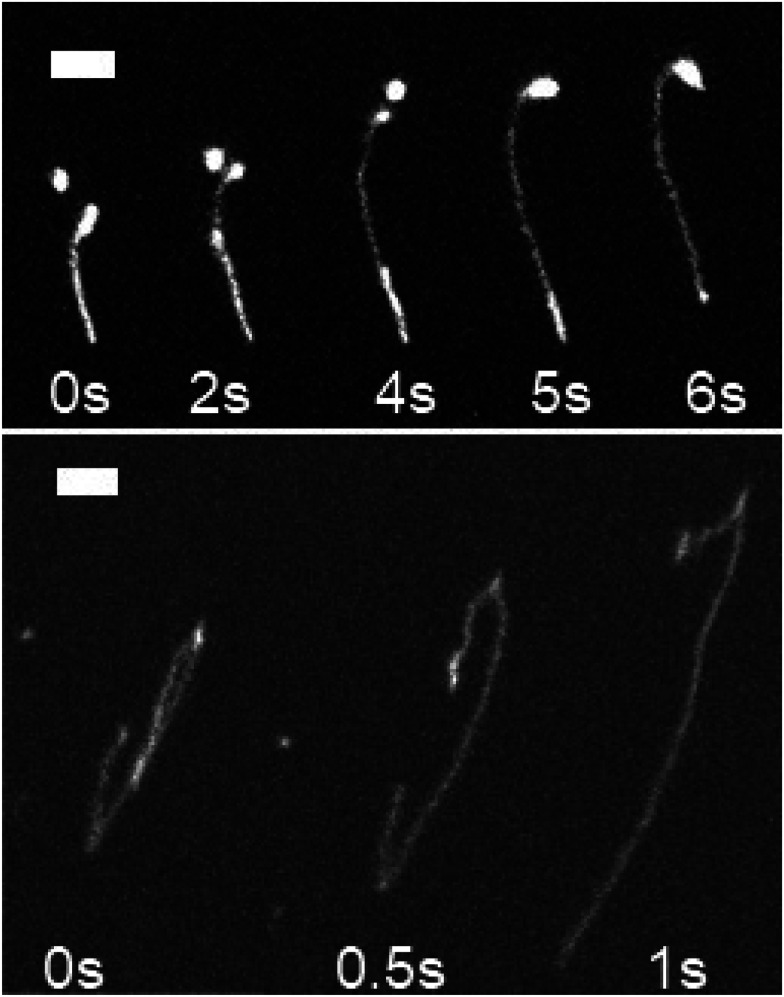
Hairpins along T4 DNA in nematic fd virus solution after shear. (Top) A DNA molecule with a ≈180° hairpin unfolding at the bottom of the macromolecule. The bright object near the top is a second DNA molecule. Two seconds elapse between shown frames. (Bottom) A DNA molecule with two hairpins. The bottom hairpin unfolds. Half a second elapses between shown frames.

Molecules are seen with sharp bends at acute angles (Fig. 1), qualitatively different from DNA sheared in isotropic fluids.^21^Fig. 1 shows two such examples, one molecule possessing an acute backfold (Fig. 1; top) and the other having two acute hairpins (Fig. 1; bottom). Unfolding requires one arm of the hairpin to slide while maintaining the angle with the other. This indicates that the DNA hairpins are topologically protected and can only be removed when the chain end reaches the joint (or two hairpins annihilate). A complete experimental characterization of this system is a subject for future work, but these qualitative observations suggest that hairpins are characteristic features of polymers embedded in a nematically ordered background. To investigate this assertion more quantitatively, we perform simulations of the suspension of flexible polymers in nematic liquid crystalline media.

## 3 Numerical model and method

To quantitatively study the conformations and dynamics of polymers embedded in nematic background, we employ a hybrid approach of N-MPCD to simulate the nematic background and MD to simulate the polymeric inclusion. N-MPCD fully incorporates thermal fluctuations, hydrodynamic interactions and nematic orientational order of the liquid crystal (Section 3.1). MD discretizes the polymer into a linear sequence of bound beads that exchange momentum with fluid particles (Section 3.2). Additionally, polymer segments are coupled to the nematic orientation *via* a two-way coupling mechanism.

### 3.1 Nematohydrodynamic model

While many microscopic particle-based methods explicitly calculate effective pair potentials between mesogen molecules,^22–25^ mesoscopic models further abstract the interactions between mesogens. An early example is the Lebwohl–Lasher model, which models the nematic phase using nematogens fixed on a cubic lattice, with interactions between particles governed by pair potentials.^26,27^ Since then other mesoscale algorithms have been conceived to simulate liquid crystal hydrodynamics, including multi-particle collision dynamics (N-MPCD) schemes.^28^ N-MPCD employs coarse-grained collision operators to evolve the density, velocity, and orientation fields. Since the N-MPCD algorithm discretizes the nematic fluid into point-particles that interact through a stochastic, many-particle collision operator, the simulation time is reduced compared to methods that calculate the pair interactions. We build on N-MPCD's success in simulating electroconvection^29^ and colloidal liquid crystals^30–32^ to consider polymer dynamics in liquid crystalline solvents.

The nematic fluid is discretized into point particles labeled *i*. Each particle possesses a mass *m*_*i*_, position *r̲*_*i*_(*t*), velocity *v̲*_*i*_(*t*) and nematic orientation *u̲*_*i*_(*t*).^28^ The number of lines under the notation indicates the tensor rank, scalars are rank-0 tensors, vectors are rank-1 tensors, and so forth. While time is discretized into discrete time steps Δ*t*, the other quantities evolve continuously. MPCD is composed of two steps: (i) streaming and (ii) collision. In the streaming step, particle positions translate ballistically according to *r̲*_*i*_(*t* + Δ*t*) = *r̲*_*i*_(*t*) + *v̲*_*i*_(*t*)Δ*t*. During the collision step, particles interact in a coarse-grained manner *via* collision operators. These cell-based operators randomly update the velocities and orientations of the particles while ensuring conservation laws are maintained. Particles are binned into cells (labeled *c*) of size *a* containing *N*_c_(*t*) particles at any instant *t*. A random grid shift ensures Galilean invariance.^33^ Only particles in the same cell interact, and every particle within each cell participates in the interaction. The collision step itself can be divided into two phases. Firstly, momentum is stochastically exchanged. The velocity of each particle *i* in cell *c* is updated using an Andersen thermostatted collision operator 
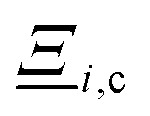
 that randomly generates velocities from a Boltzmann distribution characterized by the thermal energy *k*_B_*T*^34^ while ensuring momentum is conserved within each cell.

Secondly, each particle orientation *u̲*_*i*_, is modified by an orientation stochastic multi-particle collision operator *ψ*_c_. This operator generates new random orientations drawn from the canonical distribution of the Maier–Saupe mean-field approximation about the local director *n̲*_c_ within each MPCD cell.^28^ The orientation collision operator is characterized by a globally specified nematic interaction constant *U*, which controls how strongly the orientations align. Additionally, nematogens interact with velocity gradients in fluid flow and reorient. Further details on the collision step are included in Appendix A.

Velocity-orientation coupling is achieved by application of Jeffery theory for reorientation of particles possessing a bare tumbling parameter *λ* and shear susceptibility *α*. The heuristic parameter *α* adjusts the alignment relaxation time relative to Δ*t*, effectively allowing Jeffrey's equation to be averaged over a small number of time steps of the fluctuating hydrodynamic field (Appendix A). The bare tumbling parameter *λ* of an isolated rod is related to its aspect ratio, approaching zero for spherical particles and unity for infinitely elongated filaments.^28^ However in liquid crystals, |*λ*| > 1 is possible,^35^ in which case the nematogens align with the shear flow and the liquid crystal is said to be flow aligning. Backflow is accounted for by balancing the change in angular momentum generated by the orientational collision operator with an angular momentum conserving term in the velocity collision operator,^34^ the magnitude of which is governed by a rotational friction coefficient *γ*_R_.^28,34,36^ A small value is selected to minimize backflow effects.

The N-MPCD method allows for simulations of two and three-dimensional nematic fluids, possessing isotropic-nematic phase transitions with annihilation of defects in 2D. While the resulting nematic fluid is expected to exhibit some anisotropic viscous dissipation due to the inclusion of a tumbling parameter, subsequent numerical analysis has shown that the N-MPCD method obeys linearized nematohydrodynamics where viscosity and elastic effects are isotropic.^37^ All particles have the same mass *m*. The N-MPCD particle mass *m*, thermal energy *k*_B_*T* and cell size *a* set the simulation units, including units of time 
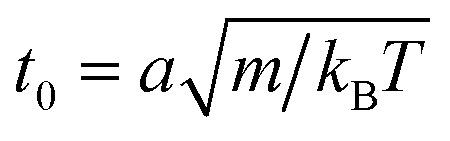
. The N-MPCD streaming time step is set to Δ*t* = 0.1*t*_0_ and the N-MPCD number density is 20/*a*^3^ giving a Schmidt number of ≈375.^38^ The N-MPCD parameters are chosen to be *γ*_R_ = 0.01*k*_B_*Tt*_0_, *λ* = 2 and *α* = 0.5. These parameters control two-way coupling between the director and fluid flow, representing flow-aligning liquid crystal.^28^ Selecting a small rotational friction minimizes the backflow and mitigates any related effects in this study. The nematic interaction constant *U* = 6*k*_B_*T* is within the nematic phase^28^ but with an energy landscape that is low enough for the polymer to explore conformational space in computationally feasible times.

### 3.2 Polymer model

The flexible polymer is simulated by molecular dynamics (MD)^39^ simulations. It is composed of *N* beads (*j* = 1,…,*N*) with mass *M*, which obey the equations of motion1
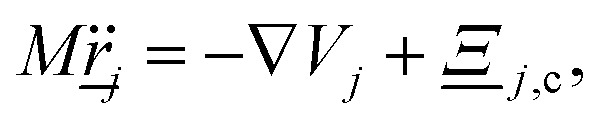
in which *V*_*j*_ is the total potential of particle *j* and 
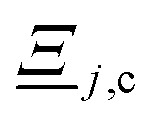
 is the thermal and hydrodynamic drag forces due to including MD particle *j* in the MPCD collision of cell *c*. The beads interact *via* pair potentials which are composed of a bond *V*_FENE_, steric effects *V*_LJ_ and nematic coupling *V*_NPC_ terms. We model freely jointed chains with no internal bending potential.

Beads are linearly connected by a finitely extensible nonlinear elastic (FENE) bond potential^40–42^2
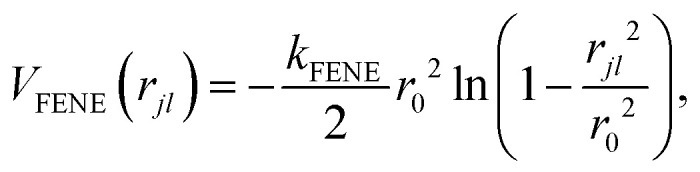
where *k*_FENE_ is the bond strength, *r*_0_ is the equilibrium length of the bonds and *r*_*jl*_ = |*r̲*_*jl*_| for *r̲*_*jl*_ = *r̲*_*j*_ − *r̲*_*l*_ between monomers *j* and *l*. For bonds (eqn (2)), *l* = *j* − 1. Excluded-volume interactions are taken into account by the purely repulsive Lennard-Jones potential^40–43^3
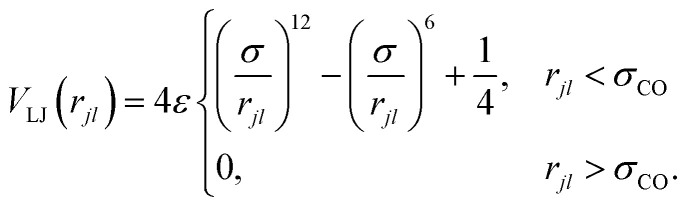
The energy *ε* sets the strength of the repulsive potential, *σ* is the effective size of a bead and 
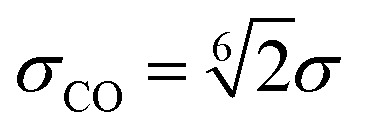
 is the cutoff.

**Fig. 2:**
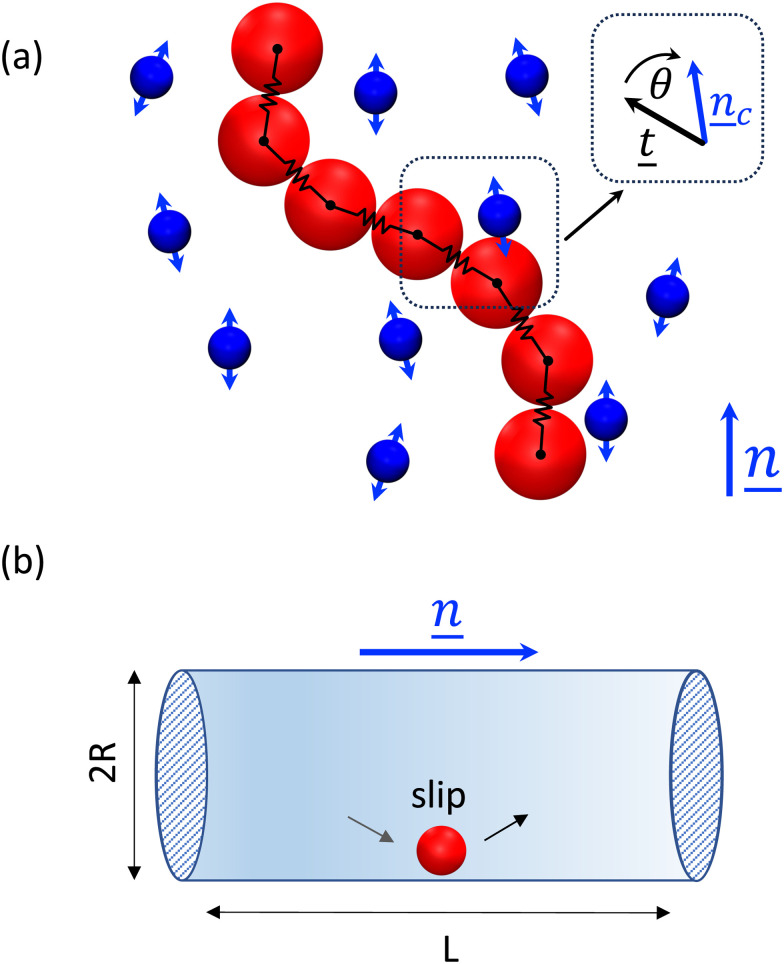
Simulation and boundary conditions. (a) The orientational coupling potential between the polymer and nematic solvent is an angular harmonic potential (eqn (4)) between the backbone tangent *t̲* and the local nematic director *n̲*_c_. (b) Cylindrical system of length *L* = 30*a* and radius *R* = 10*a*. The director *n̲* is parallel to the long axis of the cylinder, with planar anchoring. The cylinder is impermeable with hydrodynamic slip conditions and periodic boundaries at both ends.

Individual monomers are not directly coupled to the surrounding nematic as might be the case for colloids with strong surface anchoring at their surface.^36,44^ Rather, segments of the polymer are anchored to the surrounding nematic by coupling the tangent between pairs of monomers along the polymer backbone to their local nematic direction *n̲*_c_ by a harmonic potential4
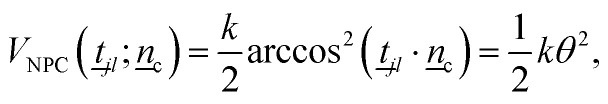
where *t̲*_*jl*_ = (*r̲*_*j*_ − *r̲*_*l*_)/*r*_*jl*_ for *l* = *j* − 1 is the tangent, *θ* is the angle between *t̲*_*jl*_ and *n̲*_c_, and *k* the coupling constant (Fig. 2a). The angular harmonic potential accounts for the tendency of polymer segments to align with the surrounding medium. This co-alignment can arise from pairwise interactions between polymer segments and nematogens, such as electrostatic dipole–dipole interactions^45^ or steric interactions^46^ (as described by the Onsager theory^47^). While eqn (4) causes segment *jl* of the polymer to rotate to align with the surrounding nematic, an equal-but-opposite torque must be applied to the local nematic to create the two-way coupling effect on the nematic. To this end, the N-MPCD mesogens within cell *c* are subject to the torque5
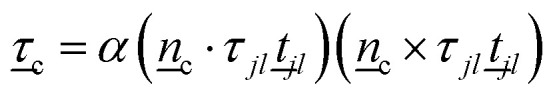
where *c* is the cell that monomer *j* resides within, and 
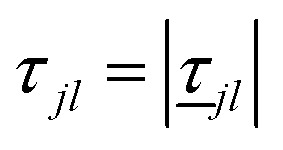
 is the torque due to *V*_NPC_ experienced by both monomers *j* and *l*. This assures that the local director feels an equal-but-opposite torque. This torque changes the orientation of local nematic mesogens by6
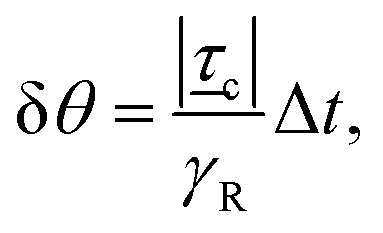
where *γ*_R_ is the rotational friction coefficient. This introduces a two-way coupling through which the polymer conformation affects the local nematic orientation. Through this coupling, the liquid crystal orientation around a polymer can be perturbed to align with the polymer (Video S1, ESI†). Likewise, the polymer backbone and nematic orientation may align at most points but a sudden bending of the polymer backbone can locally disturb the nematic order (Video S2, ESI†). While all quantitatively analyzed data is from 3D simulations (Section 3.3), we present movies of 2D simulations to illustrate this two-way coupling. These 2D simulations allow the director configurations to be clearly visible without any obstruction from the third dimension. These 2D simulations are performed in square, periodic domains of size *L* = 30*a*, with degree of polymerization *N* = 20 and coupling *k* = 20 *k*_B_*T*.

In our simulations, parameters are set to *ε* = 1*k*_B_*T*, *σ* = 1*a* and monomer mass *M* = 10*m* for all *j*. The equilibrium bond length is *r*_0_ = 1*a*, with a bond strength *k*_FENE_ = 120*k*_B_*T*/*a*^2^ and the coupling coefficients are varied from *k* = 0 to 20*k*_B_*T*. The degree of polymerization is *N* = 20, giving a contour length 19*b*, where *b* = (0.89 ± 0.02)*a* is the average bond length. The MD algorithm time step is Δ*t*_MD_ = 0.002*t*_0_, requiring 50 MD iterations per N-MPCD iteration. A summary of all variables used in the numerical model is presented in Table 1.

**Table 1:** Variables used in the numerical model

Parameter	Description	Units	Value
*a*	MPCD cell size, unit length	[Length]	1
*k*_B_ *T*	Thermal energy, unit energy	[Energy]	1
*m*	MPCD particle mass, unit mass	[Mass]	1
*t*_0_	Derived unit of time	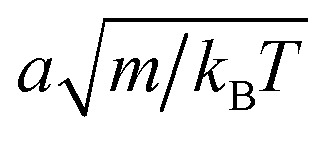	1
*U*	Inter-molecular orientation interaction strength	*k*_B_ *T*	6.0
Δ*t*	Streaming step duration	*t*_0_	0.1
*r̲*	Particle position	*a*	—
*v̲*	Particle velocity	*a*/*t*_0_	—
*u̲*	Particle orientation	—	—
*n̲*	Director	—	—
*λ*	Bare tumbling parameter	—	2
*α*	Shear susceptibility	—	0.5
*γ*_R_	Rotational friction	*k*_B_ *Tt*_0_	0.01
*N*	Degree of polymerization	—	20
*i*, *j*, *l*	Particle indices	—	—
*c*	Cell index	—	—
*M*	Monomer mass	*m*	10
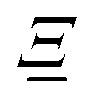	MPCD collision operator	—	—
*V*	Total potential of a monomer	*k*_B_ *T*	—
*V*_FENE_	Finitely extensible nonlinear elastic (FENE) potential	*k*_B_ *T*	—
*k*_FENE_	FENE strength	*k*_B_ *T*/*a*^2^	120
*r*_0_	FENE equilibrium bond length	*a*	1
*V*_LJ_	Repulsive Lennard-Jones (LJ) potential	*k*_B_ *T*	—
*σ*	Monomer diameter	*a*	1
*ε*	LJ strength	*k*_B_ *T*	1
*σ*_CO_	LJ cutoff distance	*a*	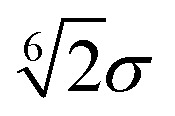
*V*_NPC_	Nematic–polymer coupling potential	*k*_B_ *T*	—
*t̲*	Monomer–monomer tangent	—	—
*k*	Nematic–polymer coupling constant	*k*_B_ *T*	(0,…,20)
*θ*	Angle between polymer tangent and local director	[Radians]	—
*τ*	Torque	*k*_B_ *T*	—
δ*θ*	Change in nematic orientation	[Radians]	—
*b*	Average bond length	*a*	0.89 ± 0.02
Δ*t*_MD_	MD time step duration	*t*_0_	0.002
*L*	System length	*a*	30
*R*	Confining cylinder radius	*a*	10

### 3.3 System setup

The simulations are conducted in a 3D cylinder of length *L* = 30*a* and radius *R* = 10*a* (Fig. 2b). Strong planar anchoring at the cylinder surface assures the global liquid crystal orientation is parallel to the long axis of the cylinder.^44^ A perfect-slip boundary condition on the impermeable cylinder wall is applied to velocity by reflecting the normal component of the velocity relative to the surface and leaving the tangential component unchanged. To allow free flow at the wall, phantom particles^48–51^ are not included. Choosing perfect-slip over no-slip assures the mobility of the polymer is not significantly affected by the presence of the wall. Periodic boundaries cap both ends of the cylinder.

The fluid is initialized with Maxwell–Boltzmann distributed speeds and the director field parallel to the cylinder axis. The polymer is initiated in the fully extended conformation on the center line of the cylinder, aligned with the global nematic direction and allowed to relax. Data is recorded once the system reaches its steady state, which is identified *via* an iterative procedure. In each iteration, the ensemble average is compared to the overall average calculated from all repetitions to find the first instance where it falls below this overall average. This time is then used as the starting point for updating the overall average. The process is repeated until successive updates of this reference time do not alter and stabilize, indicating that the system has achieved a steady state. Twenty repeats for each set of parameters are simulated, each lasting 1.2–1.5 × 10^5^*t*_0_. All simulations are performed using a custom-developed N-MPCD/MD solver.

## 4 Results

First we present how conformational properties of the polymer are affected by different coupling parameters *k*. We then show how these configurations contribute to different diffusivity of the polymer. Finally, we quantify the dynamics of hairpins. Table 2 provides a summary of all variables utilized in this section.

**Table 2:** Variables used in results

Parameter	Description	Units	Value
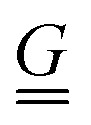	Gyration tensor of polymer	*a* ^2^	—
*r̲*_cm_	Polymer center of mass position	*a*	—
*R*_g_	Radius of gyration	*a*	—
*r*_g_	Normalized radius of gyration	—	—
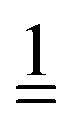	Identity rank-2 tensor	—	—
*β* = *R*_g,⊥_/*R*_g,‖_	Aspect ratio	—	—
*R̲*_e_	End-to-end vector	*a*	—
*ρ* = *R*_e_/*N*^*ν*^*b*	Normalized end-to-end distance	—	—
*ν*	Scaling exponent of polymer	—	3/5 (SAW), 1 (rod)
*Z*_1_	Partition function for a polymer segment	—	—
*ϕ*,*θ*	Azimuthal and polar angles	[Radians]	[0,2π], [0,π]
𝒦 = −*kb*/(2*k*_B_*T*)	Dimensionless coupling		
*N*_H_	Number of hairpins	—	[0,13]
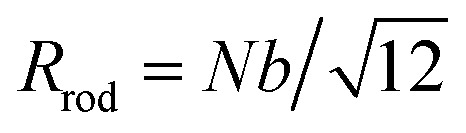	Radius of gyration for a rod with *N* monomers	*a*	—
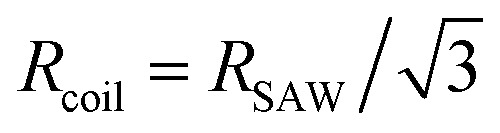	Contribution of one component to the radius of gyration for a self-avoiding random walk (SAW)	*a*	—
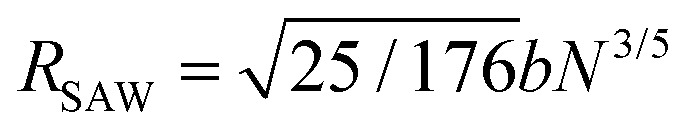	Radius of gyration for SAW	*a*	—
*χ* ^2^	Reduced chi-squared	—	—
*d*	Dimensionality	—	[2,3]
*D*	Diffusion coefficient	*a* ^2^/*t*_0_	—
*D*_H_	Hairpin diffusion coefficient	*a* ^2^/*t*_0_	—
*D*_0_	Isotropic polymer center of mass diffusion coefficient	*a* ^2^/*t*_0_	(300 ± 6) × 10^−5^
*D* ^e^	Polymer diffusion coefficient based on ellipsoidal model	*a* ^2^/*t*_0_	—
δ*t*	Lag time	*t*_0_	—
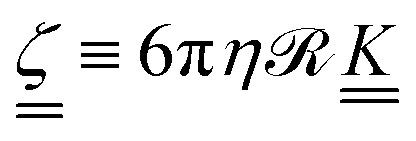	Drag coefficient	*k*_B_ *Tt*_0_/*a*^2^	—
*η*	Fluid viscosity	*k*_B_ *Tt*_0_/*a*^3^	8.6 ± 1.0
ℛ	Characteristic size of a solute	*a*	—
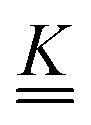	Dimensionless resistance tensor	—	—
*C*_‖,⊥_	Wall-induced correction parameters	—	1.20 ± 0.01, 1.35 ± 0.02
*Γ*	Hairpin hopping rate	1/*t*_0_	—
*E*_b_	Barrier energy	*k*_B_ *T*	—
*V*′′	Second derivative of potential with respect to angle *θ*	*k*_B_ *T*	—

### 4.1 Conformations

The average shape of the polymers can be characterized by the gyration tensor7
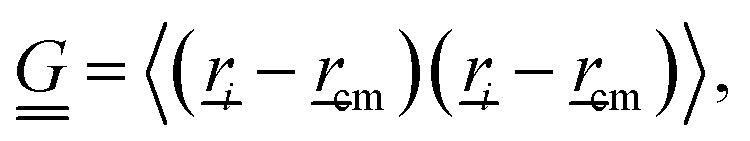
which measures the distribution of monomers around the center of mass, *r̲*_cm_ = 〈*r̲*_*i*_〉. The average 〈·〉 is over the *N* monomers. To understand how the nematic solvent impacts the conformation, we compare the values of 
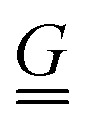
 parallel and perpendicular to the global nematic orientation *n̲*.

**Fig. 3:**
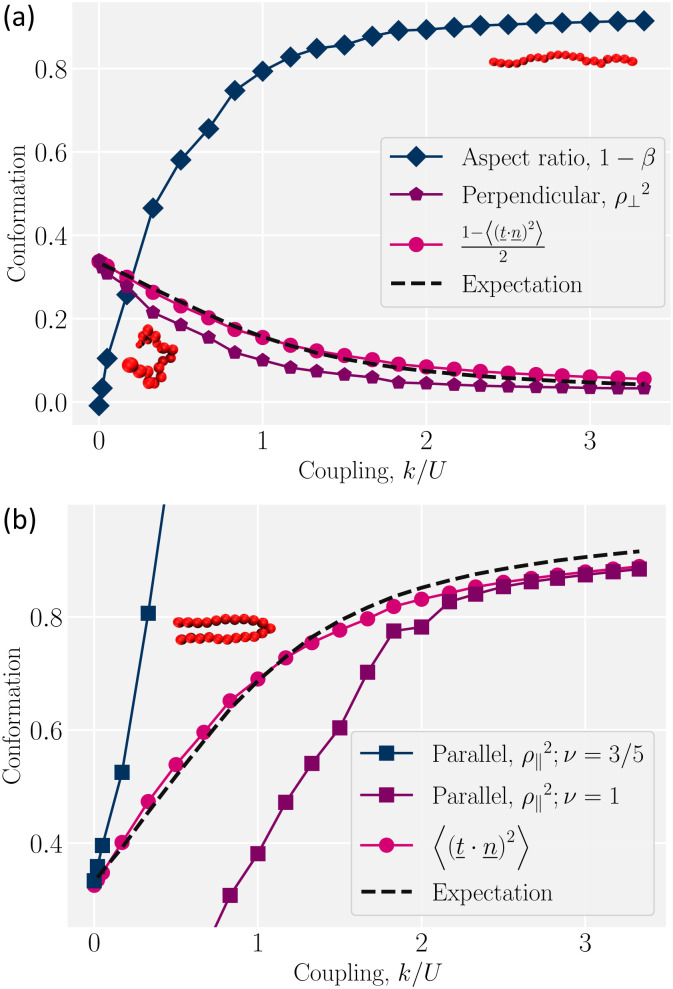
Coil-to-rod transition of a polymer as a function of the coupling to the local nematic order *k* scaled by the nematic interaction constant *U*. (a) The aspect ratio is plotted as 1 − *β* = 1 − *R*_g⊥_/*R*_g‖_. The normalized perpendicular end-to-end distance is *ρ*_⊥_^2^ = *R*_e,⊥_^2^/*N*^2*ν*^*b*^2^ with *ν* = 3/5 for self-avoiding random walk. The average of tangent perpendicular to the nematic direction (1 − 〈(*t̲*·*n̲*)^2^〉)/2 for simulations and the thermodynamic expectation value. The bottom left and upper right snapshots illustrate typical coil-like and elongated conformations, respectively. (b) The parallel end-to-end distance *R*_e,‖_^2^ is normalized by different scalings, *ρ*_‖_^2^ = *R*_e,‖_^2^/*N*^2*ν*^*b*^2^. At low coupling, *ν* = 3/5 but *ν* = 1 for a rod at high coupling. The average of tangent along the nematic direction 〈(*t̲*·*n̲*)^2^〉 is compared to the thermodynamic expectation value. The snapshot shows a single hairpin.

Due to the strong anchoring, the global director lies along the axis of the cylinder. The parallel component of the gyration tensor is 
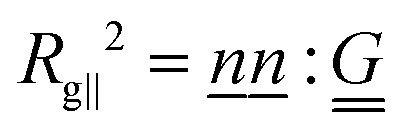
 and perpendicular 
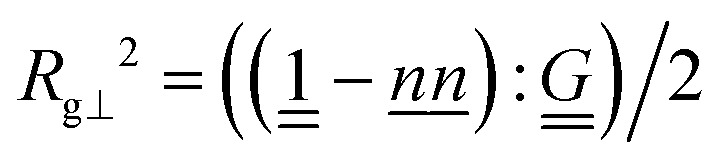
, where the double-dot product “:” is the double contraction of tensors and 
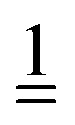
 is the identity rank-2 tensor. These form the semi-major and minor axes of an ellipsoid approximating the polymer. These values give the aspect ratio *β* = *R*_g⊥_/*R*_g‖_, which measures the degree to which the polymer is elongated. When the polymer is weakly coupled to the nematic orientation (*k*/*U* ≪ 1), it conserves its symmetry, taking an isotropic shape with 1 − *β* ≈ 0 (Fig. 3a; diamonds). As the coupling strength increases, the energy cost of misaligning with the surrounding nematic rises and causes the polymer to elongate in the nematic direction. For high coupling (*k*/*U* ≫ 1), the polymer forms a rod-like conformation with 1 − *β* ≈ 1 (Fig. 3a; diamonds). Coupling to the nematic orientation field elongates the polymer.

To understand how the aspect ratio increases, let us assume a model in which the backbone of the polymer perpendicular to the global nematic director does a self-avoiding random walk. This can be seen by considering the components of the end-to-end vector *R̲*_e_ = 〈*r̲*_N_ − *r̲*_1_〉. The end-to-end distance of the perpendicular random walk 
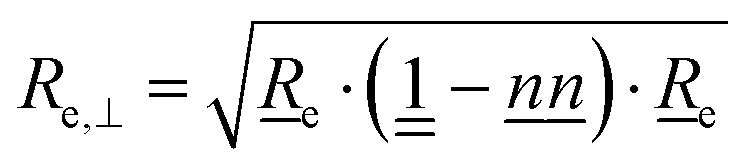
 scales with the number of steps as *R*_e,⊥_ ∼ *N*^*ν*^, where *ν* = 3/5. To compare this prediction to the simulations, we measure the perpendicular end-to-end distance and normalize it as *ρ*_⊥_ = *R*_e,⊥_/*N*^*ν*^*b* for *ν* = 3/5 (Fig. 3a; pentagons). If the tangent *t̲* is decomposed into parallel *t̲*_‖_ = *n̲n̲*·*t̲* and perpendicular 
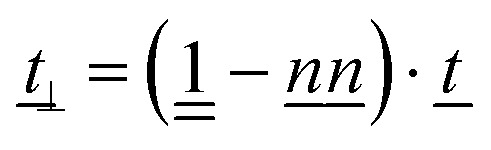
 components, the size of the steps in the perpendicular direction is 
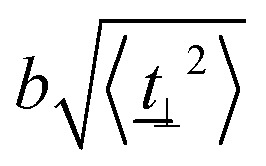
. Thus, we ideally expect *ρ*_⊥_^2^ = 〈*t̲*_⊥_^2^〉. At the low coupling limit (*k*/*U* ≪ 1), *ρ*_⊥_^2^ = 1/3 is expected for a coil-like random walk because the bond tangent vector *t̲* is a unit vector whose average in each direction is isotropic and so each component contributes 1/3. At high coupling (*k*/*U* ≫ 1), *ρ*_⊥_^2^ gradually goes to zero (Fig. 3a; pentagons) as expected from the decreased degrees of freedom in the perpendicular direction and the suppression of 〈*t̲*_⊥_^2^〉.

To further explore the role of the perpendicular fluctuations, we measure them directly from simulations as 〈*t̲*_⊥_^2^〉 = (1 − 〈(*t̲*·*n̲*)^2^〉)/2 (Fig. 3a; circles). The normalized end-to-end distance *ρ*_⊥_^2^ qualitatively agrees with 〈*t̲*_⊥_^2^〉. However, there are quantitative differences, especially at intermediate couplings. We hypothesize that the difference is primarily due to the finite size of the polymer and build an analytical model for the large-*N* thermodynamic limit.

Assuming each segment fluctuates independently, the partition function – the sum over all possible states – of a single segment in the continuum limit is 
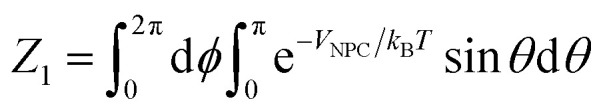
. The form of the aligning harmonic potential used in the simulations, *V*_NPC_ = *kθ*^2^/2 (eqn (4)), does not permit an analytical solution for the partition function. Therefore, we adopt the approximation *V*_NPC_ ≈ *k* sin^2^ *θ*/2 = *k*(1 − (*t̲*·*n̲*)^2^), which similarly penalizes deviations from the local nematic orientation and has been used in previous studies.^13,52^ With this approximation, the partition function of a single segment becomes8
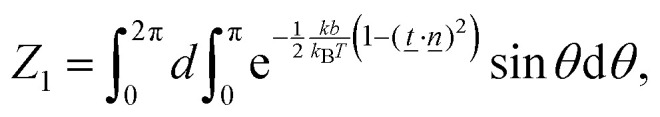
which involves integrating over the azimuthal *ϕ* and polar *θ* angles. The integral over the unit sphere gives9
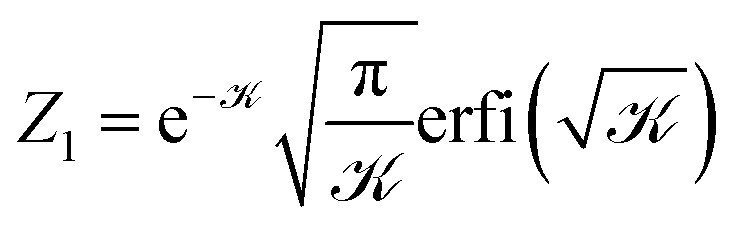
with the dimensionless coupling 𝒦 = −*kb*/(2*k*_B_*T*).

We differentiate the free energy of one bond (−*k*_B_*T* ln *Z*_1_) with respect to the coupling parameter *k* to obtain the expectation value for its thermodynamic conjugate10
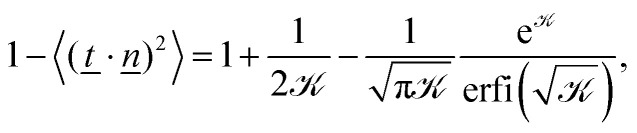
which is equivalent to 2〈*t̲*_⊥_^2^〉 (Fig. 3a; dashed line). The thermodynamic expectation value agrees well with the perpendicular tangent 〈*t̲*_⊥_^2^〉 but only qualitatively with the normalized end-to-end distance in the perpendicular direction *ρ*_⊥_^2^. This leads us to conclude that, even though individual segments are in equilibrium and fluctuating about the global nematic director, the overall conformation is more complex than a simple sum of these fluctuations. As we will show in Section 4.2, this is due to the existence of hairpins.

A similar line of argument follows for the parallel direction. Similar to the perpendicular component, the thermodynamic expectation value11
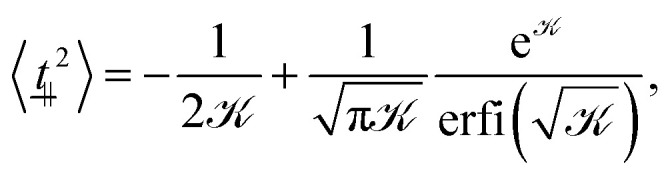
agrees well with simulation results for all coupling parameters (Fig. 3b; dashed line and circles, respectively). The parallel component of the end-to-end vector *R*_e,‖_^2^ = *R̲*_e_·*n̲ n̲*·*R̲*_e_ is more complicated. At low coupling (*k*/*U* ≪ 1), the polymer performs a self-avoiding random walk with the normalized parallel end-to-end distance *ρ*_‖_^2^ = *R*_e,‖_^2^/*N*^2*ν*^*b*^2^ = 〈*t̲*_‖_^2^〉 = 1/3 with *ν* = 3/5 (Fig. 3b; blue squares).

While the theory and simulations agree for the limit of very low coupling, the normalized end-to-end distance *ρ*_‖_^2^ quickly diverges and deviates from the parallel tangent. This is because the assumption of *ν* = 3/5 for an isotropic coil breaks down. Likewise, at high coupling (*k*/*U* ≫ 1), the polymer is a rod *ρ*_‖_^2^ = *R*_e,‖_^2^/*N*^2*ν*^*b*^2^ → 1 with *ν* = 1 in this limit (Fig. 3b; purple squares). The theory fails to reproduce the parallel extension *ρ*_‖_^2^ for intermediate coupling in the parallel direction due to crossing between scalings. This is due to the formation of hairpins – sudden turns along the backbone of the polymer (Fig. 3b, snapshot of the polymer).

### 4.2 Hairpins

Hairpins arise in polymers suspended in nematic fluids because of the competition between conformational entropy and the energy due to being coupled to the nematic background.^52^

For weak coupling between the polymer and the nematic (*k*/*U* ≪ 1), entropy maximization is dominant and polymers randomly explore conformational space (Fig. 3a; low coupling snapshot). On the other hand for strong coupling (*k*/*U* ≫ 1), the energy cost of misaligning with the surrounding liquid crystal is so high that the elongated conformations are preferred (Fig. 3a; high coupling snapshot). However, at intermediate coupling neither contribution to the free energy is negligible. The polymer forms hairpin-like conformations to simultaneously satisfy the nematic symmetry and retain access to many conformational states. The resulting hairpins are localized to sudden turns so that the energy cost of a hairpin is not substantial (Video S2, ESI†). Because these hairpins can diffuse along the backbone of the polymer, the polymer can access many conformations with the same energy, allowing the entropy gain to compensate the energy cost (Videos S3 and S4, ESI†).

**Fig. 4:**
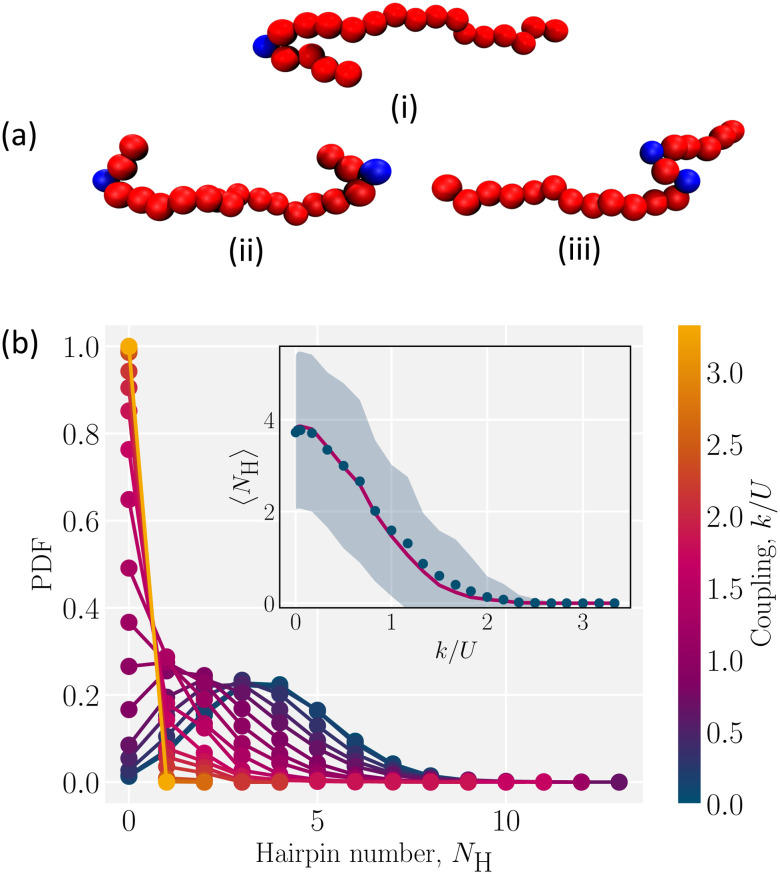
Hairpin formation and distribution. (a) Snapshots of different types of hairpin formation due to thermal fluctuations (i) single hairpin at one end (ii) two hairpins at both ends (iii) two hairpins (pair creation) away from ends. (b) Probability distribution function (PDF) of number of hairpins *N*_H_, for different coupling parameters *k*. (Inset) The measured average (dots) compared to the fit (solid line) of the PDF for each coupling to a Poisson distribution. The shaded region is the standard deviation.

Hairpins are formed by thermal fluctuations that are large enough to let the polymer overcome the elastic barrier of the nematic liquid crystal. They form either as single hairpins forming at either ends (Fig. 4a; i and ii, Video S3, ESI†) or as pairs at any point along the backbone (Fig. 4a; iii, Video S3, ESI†). By qualitative comparison of the results of simulations with experiments, we see that the single hairpin example (Fig. 4a; i) is similar to top panel of Fig. 1 from experiments and the two hairpins case (Fig. 4a; ii) is similar to bottom panel of Fig. 1.

To identify hairpins, each monomer is given a “hairpin score”, which measures the different features of hairpins. Monomers that have a sufficiently high score are identified as hairpins (Appendix B for details). Based on this identification, we measure the number of hairpins for different coupling. For the low coupling (*k*/*U* ≪ 1), the probability distribution function (PDF) of the number of hairpins *N*_H_ is wide (Fig. 4b). The distribution has a maximum of 13 “hairpins”. In this coil-like configuration, identifying these as hairpins is not particularly meaningful since the self-avoiding random walk has many sudden turns that are not related to the director orientation.

As the coupling increases, the energy barrier rises and the likelihood of hairpin formation decreases. This causes the PDFs to shift to lower values and narrow. The resulting lower average number of hairpins 〈*N*_H_〉 (Fig. 4b; inset) has a smaller standard deviation than the weak coupling limit (Fig. 4b; shaded area in inset). The elastic nature of the nematic solvent dominates the thermal fluctuations when *k*/*U* ≫ 1 and 〈*N*_H_〉 → 0 (Fig. 4(b); inset). The number of hairpins are Poisson distributed, which is shown by comparing the measured average number of hairpins to the Poisson fit of the PDF (Fig. 4b; inset). This suggests that hairpins form randomly and independently.

**Fig. 5:**
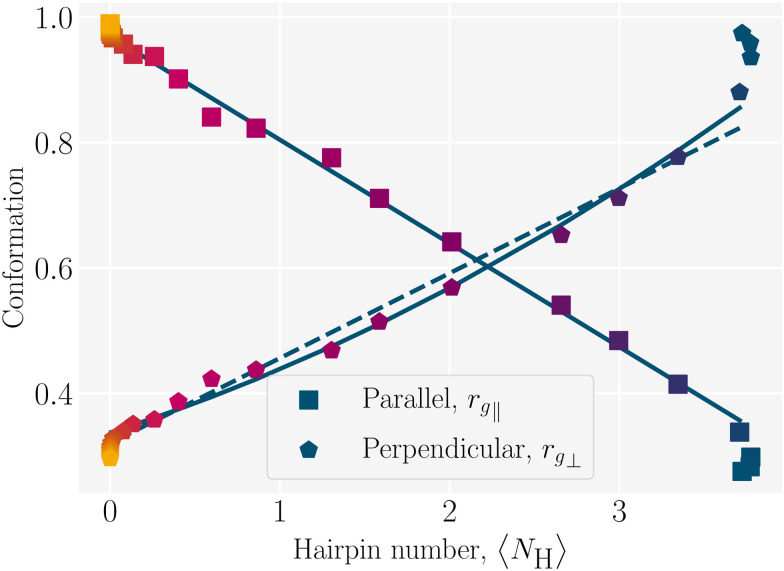
Conformation as a function of hairpin number *N*_H_. The normalized parallel component of the radius of gyration is *r*_g‖_ = *R*_g‖_/*R*_rod_, where *R*_rod_ is the radius of gyration for a rod. The perpendicular component of gyration radius *r*_g⊥_ = *R*_g⊥_/*R*_coil_ is normalized by the perpendicular component of gyration radius for a self-avoiding polymer. At low hairpin number (〈*N*_H_〉 → 0), the conformation is rod-like with *r*_g‖_ → 1. At high hairpin number (〈*N*_H_〉 ≳ 3), the conformation is a coil with *r*_g⊥_ → 1. The primary fits are plotted as solid lines, linear for the parallel component and quadratic for perpendicular component. A linear fit to the perpendicular component is plotted as a dashed line. The colors correspond to the color bar in Fig. 4.

We previously demonstrated that the conformation is well predicted by theory in the low and high coupling limits, but hairpins strongly modify the observed conformations at intermediate couplings (Section 4.1). Having quantified the number of hairpins, the conformational properties at intermediate couplings can be explored as functions of number of hairpins. For this purpose, we return to the parallel and perpendicular components of gyration tensor. The normalized parallel component of gyration tensor is *r*_g‖_ = *R*_g‖_/*R*_rod_, where 
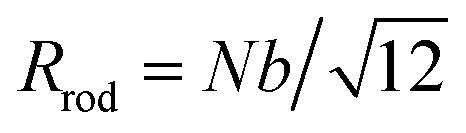
 is the gyration radius for a rod with *N* monomers connected by bonds of length *b*. The normalized perpendicular component of gyration tensor is *r*_g⊥_ = *R*_g⊥_/*R*_coil_, where 
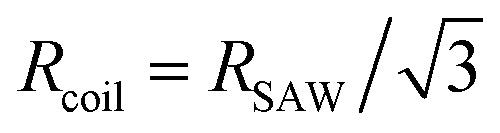
 is the contribution due to one component of the gyration radius for a self-avoiding polymer 
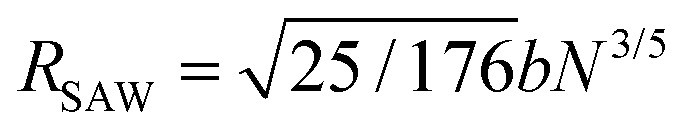
 (Fig. 5; symbols).^53^ In the parallel direction, the gyration radius linearly decreases with average number of hairpins, *r*_g‖_ = (−0.165 ± 0.003)〈*N*_H_〉 + (0.969 ± 0.005) (Fig. 5; solid line). The linear dependence of *r*_g‖_ on 〈*N*_H_〉 highlights the significance of hairpins in the adopted shape of the polymers. Since strong coupling results in no hairpins and weak coupling results in many, it is the intermediate region of Fig. 5 that is of primary interest and the fits are performed over the range 0.01 ≤ 〈*N*_H_〉 ≤ 3.7.

Similarly, the perpendicular component exhibits a linear dependence, *r*_g⊥_ = (0.135 ± 0.005)〈*N*_H_〉 + (0.320 ± 0.009) (Fig. 5; dashed line). While a linear fit is good, including a quadratic term *r*_g⊥_ = (0.337 ± 0.007) + (0.086 ± 0.012)〈*N*_H_〉 + (0.014 ± 0.003)〈*N*_H_〉^2^ further improves the agreement (Fig. 5; solid line). The goodness of these fits are assessed using the reduced chi-squared *χ*^2^. The values of *χ*^2^ = 0.33 for the linear and *χ*^2^ = 0.20 for the quadratic fit indicate that both are acceptable but that including the quadratic term better represents the results.

### 4.3 Dynamics

#### 4.3.1 Center of mass dynamics

The hairpins’ impact on polymer dynamics can be seen by focusing on the polymer center-of-mass dynamics. The dynamics of polymer center of mass is characterized by its mean squared displacement (MSD)

12 〈δ*r̲*_cm_^2^〉 = 2*dD*δ*t*,

where δ*r̲*_cm_ = *r̲*_cm_(*t* + δ*t*) − *r̲*_cm_(*t*). From eqn (12), the isotropic diffusion coefficient *D* is determined for the dimensionality *d*. However, to understand how the nematic solvent affects the diffusion of the center of mass of the polymer, the MSD is decomposed into parallel and perpendicular components

13 〈δ*r̲*_cm_·*n̲ n̲*·δ*r̲*_cm_〉 = 2*dD*_‖_δ*t*

14
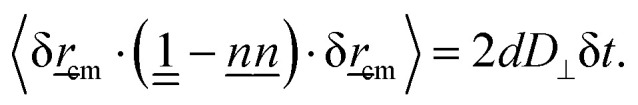


**Fig. 6:**
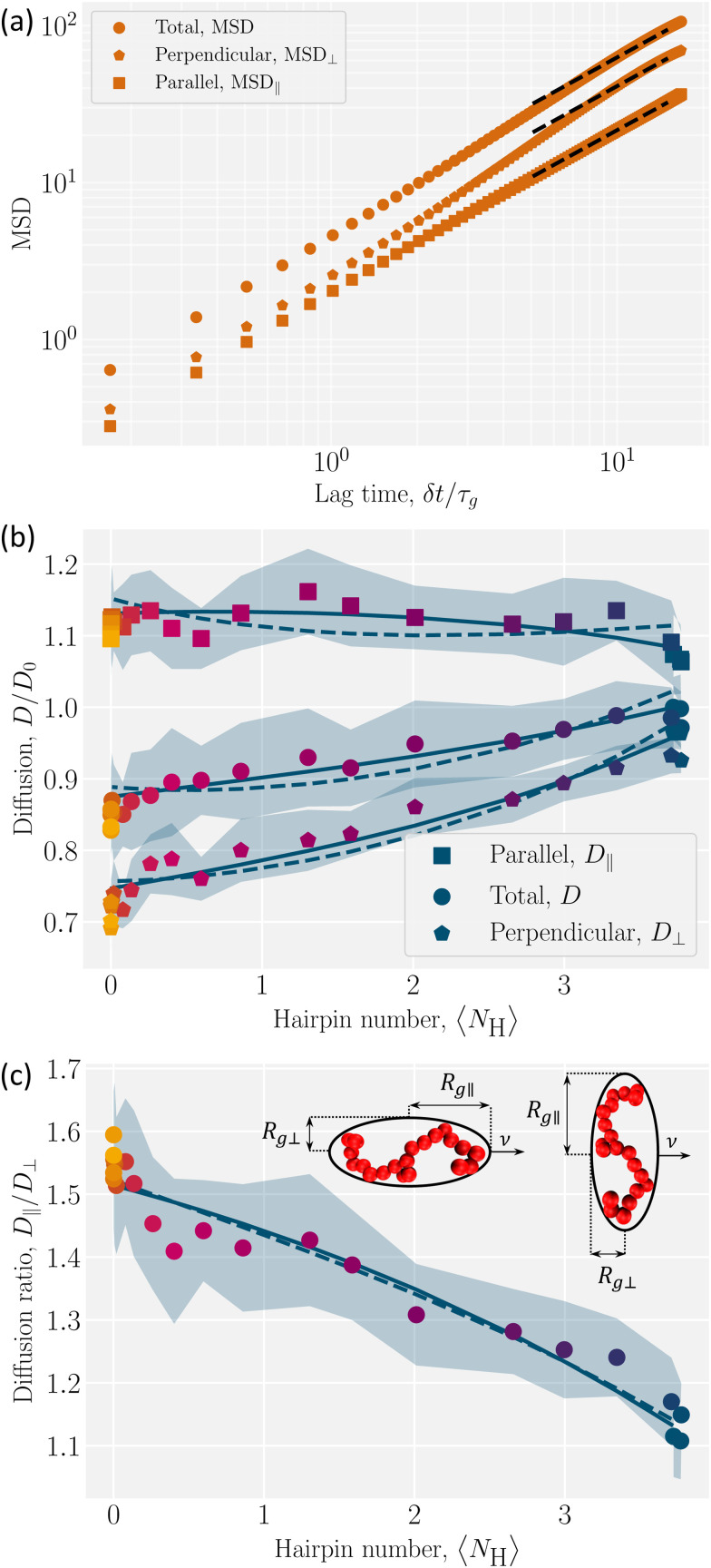
Diffusion of polymer center of mass of as a function of hairpins number. (a) An example MSD with its parallel (MSD_‖_) and perpendicular (MSD_⊥_) components for *k*/*U* = 2.5 with 〈*N*_H_〉 = 0. The lag time is normalized by *τ*_g_, the time for the uncoupled (*k* = 0) polymer to diffuse its size *R*_g_. The dashed lines show the fits. (b) Total *D*, parallel *D*_‖_ and perpendicular *D*_⊥_ diffusivities are normalized by *D*_0_, the uncoupled diffusion coefficient. (c) The ratio of parallel to perpendicular diffusivity. (Inset) Ellipsoids of semi-major (*R*_g‖_) and semi-minor (*R*_g⊥_) axes, with different orientations to the direction of motion *v̲*. In (b) and (c), solid lines show the ellipsoidal model, where the parallel gyration radius is fitted linearly and the perpendicular component quadratically. The dashed lines are for the linear fit of the perpendicular component. Shaded regions shows the standard deviations and colors correspond to the color bar in Fig. 4.

An example MSD for coupling parameter *k*/*U* = 2.5 is plotted with its components in Fig. 6a. Eqn (13) and (14) with *d* = 1 and *d* = 2 give the parallel *D*_‖_ and the perpendicular *D*_⊥_ diffusion coefficients, respectively. While the parallel diffusion stays unchanged as the average number of hairpins 〈*N*_H_〉 increases with decreasing coupling, the perpendicular and total diffusion increase as 〈*N*_H_〉 increases (Fig. 6b).

To understand this anisotropy, consider two possible sources of diffusion anisotropy in liquid crystalline solvents. The first is the anisotropy in the viscosity of the solvent. In nematic solvents viscosity is lower along the nematic director and it has been shown that spheres diffuse anisotropically in a nematic solvent, with *D*_‖_/*D*_⊥_ ≈ 1.6.^54,55^ The second source of the anisotropy is the shape of the solute. For instance, for a rod-like inclusion, diffusion along the axis of the rod is less hindered than that in the direction perpendicular to the rod axis. For an infinitely long thin rod in an isotropic solvent *D*_‖_/*D*_⊥_ = 2.^56^ Since our nematic liquid crystal does not have anisotropic viscosity,^37^ the reason behind the measured anisotropic diffusion must be the anisotropy in the shape of the solute. However, due to the finite length of the polymer, the ratio *D*_‖_/*D*_⊥_ converges to approximately 1.6, which is less than 2.

The diffusion coefficients are related to the drag coefficients through the Einstein relation as 
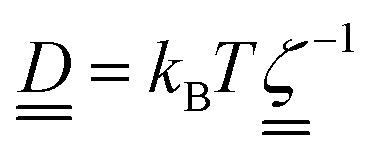
 (ref. 57) and so the anisotropy in the shape of the solute affects its diffusivity through anisotripic drag coefficients. The drag coefficients15
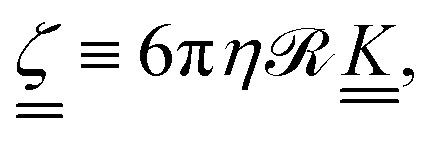
are related to the fluid viscosity *η*, the characteristic size of the solute ℛ and its shape, accounted for by dimensionless resistance tensor 
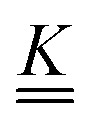
.^58^ The resistance tensor 
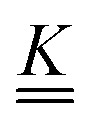
 is known for various shapes, including prolate ellipsoids (Appendix D). Prolate ellipsoids are a reasonable first-order model for the elongated conformations of polymers in nematic fluids. Approximating the polymer to first order as an ellipsoid gives the ratio of expected diffusivities for ellipsoids (denoted by superscript e) *D*^e^_‖_/*D*^e^_⊥_ as a function of only the aspect ratio *β* (Appendix D). In an isotropic fluid when the shape is symmetric (*β* = 1), *D*^e^_‖_ = *D*^e^_⊥_ and their ratio has its minimum value of unity. For the rod-like limit (*β* ≪ 1), the ratio rises to its maximum value of *D*^e^_‖_/*D*^e^_⊥_ = 2.^56^ We use this ellipsoidal model with semi-major axis *R*_g‖_ and semi-minor axis *R*_g⊥_ (Fig. 6c; inset), to confirm that anisotropy in the diffusion rises only due to shape anisotropy.

However, the polymer is inside a cylinder and the cylinder wall affects polymer diffusion. To account for the wall effect, correction factors are applied to the ellipsoidal expectation values,^59^16
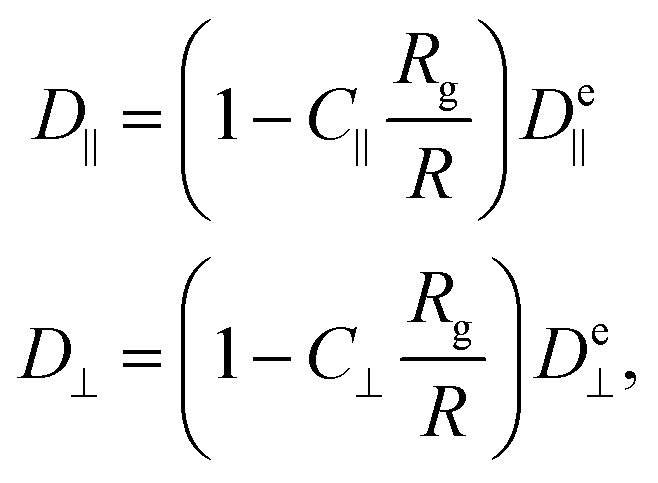
with the radius of the cylinder *R* and 
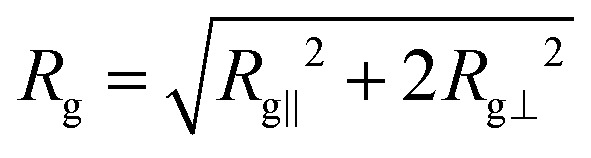
 the gyration radius in the absence of coupling (*k* = 0). Applying these corrections to the values calculated using eqn (26) reproduces the diffusion coefficients for the polymer embedded in a nematic inside the cylinder (Fig. 6b and c; solid lines). The correction parameters are fit to *C*_‖_ = 1.20 ± 0.01 and *C*_⊥_ = 1.35 ± 0.02. The diffusion coefficients demonstrate good agreement with the simulation results. The radius of gyration as a function of the number of hairpins used in eqn (16) comes from the fits shown in Fig. 5. While a linear fit to both the parallel and perpendicular gyration radius is fairly accurate, it causes the diffusion constants to be under predicted and *D*_‖_ to be predicted to be concave up (Fig. 6b; dashed lines). Including the small quadratic correction for *r*_g⊥_ causes the ellipsoidal model to be more accurate and not curve up at high hairpin numbers (Fig. 6b; solid lines).

The analysis shows that the shape is the primary factor contributing to anisotropic diffusion. The variation in diffusion coefficients in different directions can be fully explained by the conformational changes resulting from coupling. By increasing the coupling, number of hairpins decreases and polymer stretches and becomes less symmetric (Fig. 3a). The elongated polymers with few hairpins (*k*/*U* ≫ 1) experience larger drag forces perpendicular to *n̲* which results in lower perpendicular diffusion coefficients, 
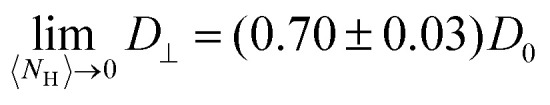
, with *D*_0_ the diffusion coefficient of the center of mass of the polymer in the absence of coupling (Fig. 6b). On the other hand, as the coupling decreases the number of hairpins increases and the shape gets more symmetric with *D*_⊥_/*D*_0_ approaching unity, 
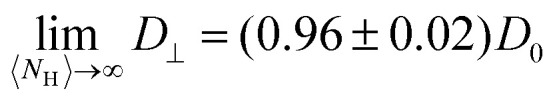
 (Fig. 6b).

In the parallel direction, diffusion starts at 
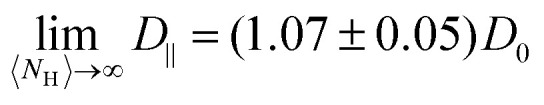
 for the limit of many hairpins and stays constant, only changing to 
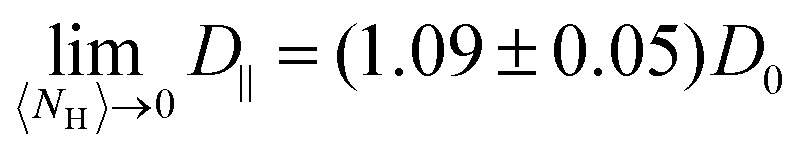
 for elongated polymers with no hairpin. Perhaps this indicates that the parallel diffusion coefficient does not vary considerably with different numbers of hairpins. However, the total diffusion coefficient *D* gradually decreases down to 
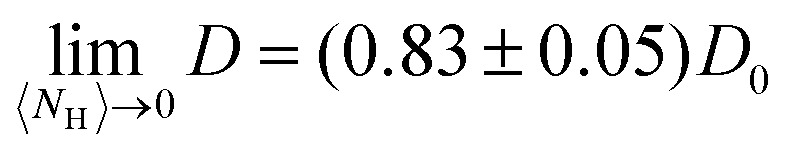
 at the strongest coupling. This should be expected since *D* = (*D*_‖_ + 2*D*_⊥_)/3, which means that the decrease in *D*_⊥_ mainly governs the total diffusion coefficient drop. We must conclude that the mobility of the polymer is primarily controlled by the effect of its conformation on perpendicular diffusivity that is caused by its coupling to the nematic orientation.

The ratio *D*_‖_/*D*_⊥_ shows a clearer comparison of the impact of coupling on diffusion coefficients in different directions (Fig. 6c). In the low coupling (*k*/*U* ≪ 1), the ratio is 
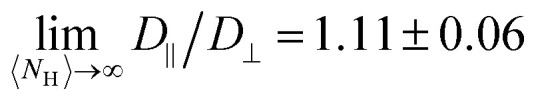
, which is close to the expected isotropic value *D*_‖_/*D*_⊥_ = 1. The deviation from unity is likely due to the cylinder wall that partially hinders the diffusion in the perpendicular direction. The ratio for no hairpins (*k*/*U* ≫ 1) is 
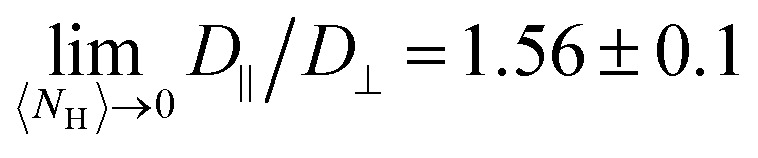
. This value is comparable to the ratio observed for a symmetric shape, such as a sphere, embedded in a nematic background with anisotropic viscosity, where *D*_‖_/*D*_⊥_ = 1.6.^54,55^ This underscores the significance of the solute's shape in its diffusivity: changes to polymer conformation should not be neglected in estimating anisotropic diffusion since their contribution is comparable to the direct contribution of anisotropic viscosity.

#### 4.3.2 Hairpin dynamics

**Fig. 7:**
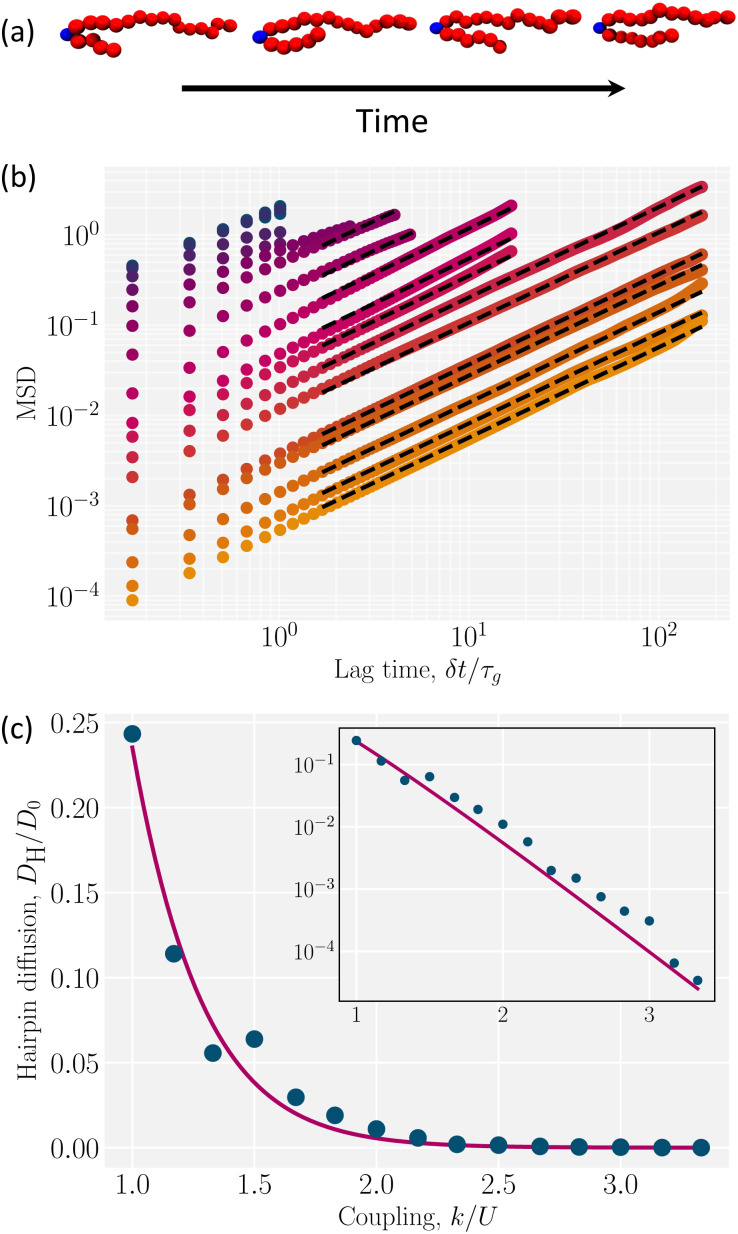
Hairpin diffusion. (a) Snapshots of hairpin diffusion. The instantaneous position of the hairpin colored in blue (Appendix B). (b) MSD examples for hairpin with various coupling parameter (the colors correspond to the color bar in Fig. 4). The lag time is normalized by *τ*_g_, the time for the uncoupled (*k* = 0) polymer to diffuse its size *R*_g_. The dashed lines represent the fit for diffusion coefficients. The colors correspond to the color bar in Fig. 4. (c) Diffusion of hairpin *D*_H_ normalized by the uncoupled polymer center-of-mass diffusion *D*_0_. The solid line represents the exponential fit and the inset shows the diffusivity on a semi-log axis.

Hairpins are the primary degree of freedom for an intermediately coupled polymer to explore its configuration space. The movement of hairpins along the polymer backbone grants polymers access to different conformations through a diffusive hopping process. We track hairpins over time (Fig. 7a, Video S4, ESI†) and use their MSD along the backbone of the polymer to measure their diffusivity for different coupling parameters *k* (Fig. 7b). Additional details on how we track the hairpins are included in the Appendix C. Because the lifetimes of hairpins are quite short for low coupling parameters (*k*/*U* < 1), the MSD does not extend to long lag times (Fig. 7b) and diffusion coefficients cannot be accurately extracted. For this reason, the analysis is restricted to *k*/*U* ≥ 1. The diffusion coefficient of hairpins decreases exponentially as the coupling parameter increases (Fig. 7c; inset).

To understand the exponential decay of the hairpin diffusion coefficient, consider the polymer backbone to be a one-dimensional lattice along which hairpins perform a hopping process (Video S4, ESI†). Each time a hairpin hops, the bond connecting its current position to the next must rotate in the director field. During this rotation, the polymer segment perturbs its surrounding liquid crystal orientation *via* the two-way coupling through *k*. This suggests there is an energy cost to overcome, which can be understood through Kramer's model of Brownian motion across a barrier of height *E*_b_.^60^ The probability current across the barrier sets the hopping rate^60^17
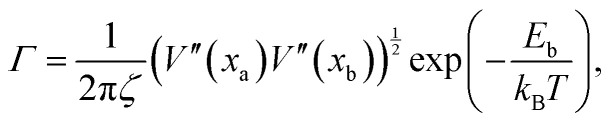
where *ζ* is a drag coefficient of the hairpin, *V*′′(*x*_a_) is the curvature of the energy well, *V*′′(*x*_b_) is the curvature of the energy barrier over which it jumps and *E*_b_ is the barrier energy.

Each of the factors in eqn (17) can be estimated (Appendix E). Substituting these estimates into eqn (17) and recognizing *D*_H_ = *b*^2^*Γ*/2 leads to the rough approximation18
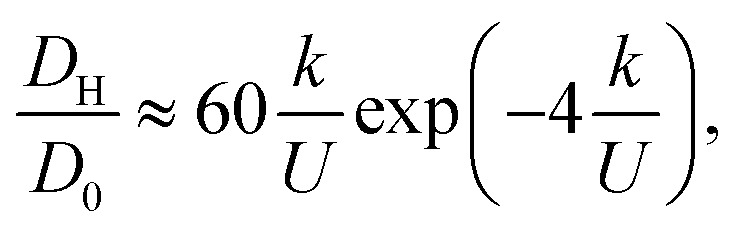
where *D*_0_ = (300 ± 6) × 10^−5^*a*^2^/*t*_0_ is the diffusion coefficient for the center-of-mass of uncoupled polymer. This rough approximation agrees within an order of magnitude with the exponential fit of the hairpin diffusion coefficient (Fig. 7c)19
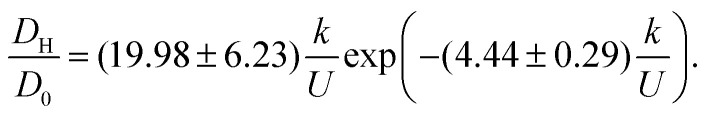
We have shown that the hairpins move diffusively by hopping over local energy barriers. In the strongly coupled limit (*k*/*U* > 1) hairpins are topologically protected defects acting as singularities for the backbone tangent vector *t̲*. One can arbitrarily designate a hairpin that opens towards the +*x̂* direction (as in Fig. 7a) to be a +1/2 hairpin (1/2 since the U-turn is 180° or half of 2π) and a hairpin that opens in the −*x̂* direction a −1/2 hairpin. As in nematic systems,^61^ defects can arise through pair creation events and be removed through pair annihilation. Additionally, individual hairpins spontaneously enter or leave the system from the polymer ends, explaining why odd *N*_H_ are observed (Fig. 4–6 and 7a).

## 5. Conclusion

This study investigated the conformation and dynamics of a single flexible polymer suspended in a nematic liquid crystal background, where its backbone is coupled to the local nematic orientation. This coupling introduces anisotropy to the polymer conformation and, if large enough, elongates the polymer. In the weak and strong limits of coupling, the extension of the polymer due to coupling can be described by the partition function for independent segments. However, for intermediate coupling, the theory fails to reproduce the observed extension values due to formation of hairpin-like configurations along the polymer. These hairpins act as topologically protected defects that minimize the internal energy by being mainly aligned with the liquid crystal and maximize entropy by moving along the polymer backbone. We quantify the number of hairpins for each coupling strength to demonstrate that polymer conformational properties are characterized by the average number of hairpins. These predictions offer a pathway for future experimental studies to indirectly measure the coupling parameter between suspended polymers and nematic solvents through polymer conformation and the distribution of the number of hairpins.

The coupling leads to anisotropic diffusion of the polymer center of mass, mainly affecting the diffusivity in the perpendicular direction. Since our nematic fluid has isotropic viscosity, this anisotropic diffusivity arises from asymmetric polymer shapes. We employ the ellipsoidal model to incorporate this polymer shape anisotropy into its drag coefficient, which directly influences its diffusivity. Our results show a good agreement with this model, confirming the shape-related anisotropy in polymer diffusion. We demonstrate that conformational effects of freely jointed polymers can be just as significant as viscosity anisotropy. This demonstrates an independent mechanism to engineer dynamics of composite nematic/polymeric materials. We further show how tuning the coupling strength can have a profound effect on hairpins dynamics, providing a potentially powerful mechanism for controlling the temporal dynamics of polymer configurations.

## Data availability

All data needed to evaluate the conclusions in the paper are present in the paper and appendix. Code available upon polite request.

## Conflicts of interest

There are no conflicts to declare.

## Supplementary Material

SM-021-D4SM00968A-s001

SM-021-D4SM00968A-s002

SM-021-D4SM00968A-s003

SM-021-D4SM00968A-s004

SM-021-D4SM00968A-s005

## Appendices

### Appendices

#### A Nematic multi-particle collision dynamics

This study employs a nematic multi-particle collision dynamics (N-MPCD) approach, which is chosen for to its capability to simulate embedded particles in a fluctuating nematohydrodynamic background. For a list of all variables introduced in the Appendices, see Table 3.

The multi-particle collision dynamics approach consists of two steps: the streaming step and the collision step. In the steaming step, the position of each particle updates assuming ballistic motion of

20 *r̲*_*i*_(*t* + Δ*t*) = *r̲*_*i*_(*t*) + *v̲*_*i*_(*t*)Δ*t*,

where *r̲*_*i*_ is the position of the particle *i*, *t* is the current time and Δ*t* is the time step.

The collision step is composed of two phases: (1) momentum exchange and (2) orientation fluctuations.

(1) Momentum exchange. The velocity of particle *i* within cell *c* is updated as , where *v̲*^cm^_c_(*t*) = 〈*v̲*_*i*_(*t*)〉_c_ is the velocity of the cell's center of mass at time *t*, 〈·〉_c_ represents the average within the cell *c* and  is the collision operator. We assume all the fluid particles have the same mass *m*. A modified angular-momentum conserving Andersen-thermostatted collision operator is employed. In the absence of angular momentum conservation, the Andersen-thermostatted collision operator is , where the components of *v̲*^ran^_*i*_ are Gaussian random numbers with variance *k*_B_*T*/*m* and zero mean.^34,62^ Although the individual velocity of each particle is randomized, subtracting 〈*v̲*^ran^_*i*_〉_c_ assures that the center of mass velocity of the cell—and consequently the total momentum—remains unchanged.^34^ To conserve angular momentum, an additional term must be added to the collision operator to remove any angular velocity generated by the collision operation.^63^ In an isotropic system, the change in the angular momentum can rise due to the stochastically generated velocities , where *r̲*_*i*,c_ = *r̲*_*i*_ − 〈*r̲*_*i*_〉_c_ is the relative position of particle *i* with respect to the center of mass of all particles in the cell. In a nematic system, changes in the orientation will contribute additional angular velocity , which will be elaborated below. For angular-momentum conserving Andersen-thermostatted N-MPCD the collision operator is

21

where the moment of inertia tensor  is for the particles in cell *c*.

(2) Orientation fluctuations. Orientation of particle *i* updates according to *u̲*_*i*_(*t* + Δ*t*) = *ψ*_c_, where *ψ*_c_ is the nematic collision operator acting on particles in cell *c*. The collision operator acts as a rotation, altering the orientation of each N-MPCD particle over the time step Δ*t*. The reorientation process can be decomposed into a (i) stochastic contribution (δ*u̲*^ST^_*i*_/δ*t*) and (ii) a flow-induced contribution (δ*u̲*^*J*^_*i*_/δ*t*) contributions.

(a) The stochastic random orientations are drawn from the canonical distribution of the Maier–Saupe mean-field approximation *f*_ori_(*u̲*_*i*_) = *f*_0_ exp(*US*_c_(*u̲*_*i*_·*n̲*_c_)^2^/*k*_B_*T*) about the local director *n̲*_c_ with normalisation constant *f*_0_ and mean field interaction constant *U*.^28^ The local director *n̲*_c_ is the eigenvector of the cell's tensor order parameter  corresponding to the largest eigenvalue *S*_c_ for the dimensionality *d* and the identity rank-2 tensor . The largest eigenvalue corresponds to the scalar order parameter. The globally specified nematic interaction constant *U* controls the strength of alignment between the MPCD nematogens.

(b) The flow-induced changes to orientation occur because the MPCD nematogens respond to velocity gradients. This flow-induced reorientation is captured through Jefferey's equation

22

for a bare tumbling parameter *λ* and heuristic shear coupling coefficient *α* in a flow with strain rate tensor  and rotation rate tensor . The rotations of nematogens generate hydrodynamic motion known as backflow, which is accounted for by the change in angular momentum , where *γ*_R_ is a rotational friction coefficient and .

#### B Hairpin identification

To identify the hairpins, five hairpin factors are considered. Each monomer is given a score , where each *p*_*n*_ measures one of the five hairpin features. Monomers with score *P*_*j*_ > *P*_cutoff_ = 0.5 are identified as hairpins.

##### Bend aligns with nematic

measures the degree to which the unit curvature vector  for monomer *j* is aligned with the local nematic director. If the segment is relatively straight,  is orthogonal to *n̲*_c_. However, if there is a 180° turn,  is parallel to *n̲*_c_ and this factor has the maximum value of 1.

##### U-turn

*p*_2_ = (*t̲*_*jj*−1_·*t̲*_*j*+1*j*_ − 1)^2^/4 assesses the degree to which the segments after and before monomer *j* are antiparallel. For a U-turn conformation *p*_2_ is close to 1; whereas, for self-avoiding random walks, this is rarely 1.

##### Arm length

*p*_3_ measures the length of the two arms around a hairpin at index *j*. To determine the arm length, pairs of monomers a distance δ*q* away from *j* are checked to see if they are antiparallel. The arm length is the maximum δ*q* for which they are antiparallel. It then normalizes the arm lengths by the maximum possible number arm length *l*_max_ = min(*j*,*N* − 1 − *j*). Specifically, , where *Θ*(·) is the Heaviside function. This value represents the relative length of hairpin arms.

##### Relative curvature

measures the relative curvature at monomer *j* in comparison with average curvature along the polymer. The sigmoid function  caps *p*_4_ to 1. The average 〈·〉 is over the *N* monomers.

##### Persistence

The last is a measure of persistence time of a hairpin at monomer *j*. To get this factor, we first check if the monomer *j* was a hairpin in the previous time step. If it was, the time possibility of being a hairpin now *p*_*t*_ will be 1. If it was not, then we check if it was an immediate neighbor to a hairpin. If it was, it has *p*_*t*_ = 1/2. Otherwise, *p*_*t*_ = 0. Non-zero values of *p*_*t*_ add to persistence time 𝒯_*j*_ of being a hairpin at monomer *j*, 𝒯_*j*_(*t* + δ*t*) = 𝒯_*j*_(*t*) + *p*_*t*_. Conversely, when *p*_*t*_ = 0, the persistence time is halved 𝒯_*j*_(*t* + δ*t*) = 𝒯_*j*_(*t*)/2. Once again, the sigmoid function ensures values less than or equal to 1, by setting *p*_5_ = *f*(𝒯_*j*_(*t* + δ*t*)).

#### C Hairpin tracking

To track the hairpins in time, the initial hairpins are identified (Appendix B) and the position *q*(0) of each hairpin along the backbone of the polymer is recorded. The position *q* is a monomer index. In each time step *t*, a new set of hairpins are identified (Appendix B) and these are checked to see which hairpins are new and which are evolutions of pre-existing hairpins from the previous time step *t* − δ*t*. If the position of any hairpin matches the position of any hairpin from the previous time step *q*(*t*) = *q*(*t* − δ*t*) or if the hairpin is at an adjacent position *q*(*t*) = *q*(*t* − δ*t*) ± 1 then the hairpin is considered an evolution of the previously existing hairpin. If the hairpin cannot be associated to any previously existing hairpin from the previous time step, it is considered a newly created hairpin. In this way, hairpin trajectories *q*(*t*) can be tracked in time (Video S4, ESI†). From these tracks, the mean-squared displacement can be computed and the diffusion coefficient for hairpins in one dimension can be found.

#### D Ellipsoidal model of diffusion constants

The diagonalized resistance tensor in the drag (eqn (15)) for an ellipsoid with an aspect ratio *β* is

23

where

24

25

for prolate ellipsoids *β* < 1.^64^ Thus, the expected parallel and perpendicular diffusion coefficients are

26

and their ratio is

27

#### E Estimation of hairpin hopping rate

For the hairpin hopping along the polymer backbone coupled to the nematic orientation, eqn (17) gives the hopping rate *Γ* in terms of the energy landscape. It involves the effective drag of a hairpin *ζ*, barrier height *E*_b_ and the second derivatives of the potential *V*′′ at the local minimum *x*_a_ and barrier location *x*_b_. The curvature of the local potential well at its minimum is

28

simply the second derivative of eqn (4). The curvature at the barrier peak *V*′′(*x*_b_) is estimated by the maximum change in the energy *versus* the minimum change in angle

29

The maximum change in energy occurs when a bond transitions from being fully parallel to the nematic orientation to being fully perpendicular, Δ*V*_max_ = *k*(π/2)^2^/2. For the small variation in angle, the arc length is Δ*s* ≈ *b*Δ*θ*_min_ = *v*Δ*t*_MD_, which suggests Δ*θ*_min_ ≈ *v*Δ*t*_MD_/*b*, where *v* is the speed of monomers. Based on the equipartition theorem, the average energy for each degree of freedom is *k*_B_*T*/2 which leads to , in 3D. Substituting these parameters in MPCD units into eqn (29) results in

30 *V*′′(*x*_b_) ≈ 90^2^*k*.

We estimate that the drag coefficient of a hairpin is similar to the drag coefficient of a monomer *ζ* ≈ 6π*ησ* ≈ 160. Substituting all of this into eqn (17) gives *Γ* ≈ 0.09*k*e^−*E*_b_/*k*_B_*T*^.

Finally, the barrier energy *E*_b_ must be estimated. To proceed with this estimation, we assume a hairpin consists of two consecutive perpendicular bonds, each forming π/4 angle with the nematic orientation. For the hairpin to hop to its next position, these bonds must move forward or backward along the polymer backbone. The energy cost of this process is

31

These rough values can be substituted in eqn (17) to approximate the hairpin diffusion constant, and the prediction is found to be consistent with the measured diffusivity (eqn (19)).

#### F Variable lists

**Table 3:** Variables used in the appendices

Parameter	Description	Units	Value
*v̲* ^cm^	Velocity of the center of mass	*a*/*t*_0_	—
	Velocity collision operator	—	—
*v̲* ^ran^	Random velocity	*a*/*t*_0_	—
	Change in the angular momentum	*k*_B_ *Tt*_0_	—
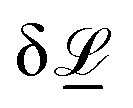
	Moment of inertia tensor	*k*_B_ *Tt*_0_^2^	—
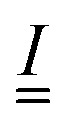
*ψ*	Nematic collision operator	—	—
δ*u̲*/δ*t*	Temporal change of orientation vector	1/*t*_0_	—
*S*	Scalar order parameter	—	[0,1]
	Tensor order parameter	—	—
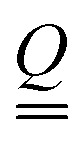
	Strain rate tensor	1/*t*_0_	—
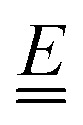
	Rotation rate tensor	1/*t*_0_	—
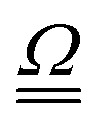
*P*	Hairpin score	—	[0,1]
*p*	Probability for hairpin features	—	[0,1]
*P*_cutoff_	Hairpin score cutoff value	—	0.5
	Curvature	—	—
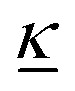
δ*q*	Distance between monomer pairs	*a*	[*b*,(*N* − 1)*b*]
*l*_max_	Maximum possible arm length for a hairpin	*a*	[1,(*N* − 1)/2]
*Θ*(*x*)	Heaviside function	—	
𝒯	Hairpin persistence time	*t*_0_	—
𝒮	Shape descriptor in ellipsoidal model	—	—
Δ*V*_max_	Maximum change of potential	*k*_B_ *T*	—
Δ*θ*_min_	Minimum change in the angle	[Radians]	—
Δ*s*	Arc length	*a*	—
