# Supplementary material for: Dynamics of polymers in coarse-grained nematic solvents
Source: Soft Matter. 2024 Nov 6;21(3):361–75. doi: 10.1039/d4sm00968a (PMC11612746; doi:10.1039/d4sm00968a)
Supplement: SM-021-D4SM00968A-s005 [file SM-021-D4SM00968A-s005.pdf]

Supplementary Information (SI) for Soft Matter.
This journal is © The Royal Society of Chemistry 2024

Video SI-1.mp4 Two-way nematic-polymer coupling. The polymer is visualized as a black line, the director as white lines and the scalar order parameter S_c as the colour map. The nematic orientation surrounding the polymer is aligned with its backbone. Although all presented quantitative results are in 3D, this simulation is carried out in 2D to visualize the coupling. The polymer consists of $N = 20$ monomers, with a coupling parameter $k = 20$, embedded in a square domain of $L = 30$ with periodic boundary conditions.

Video SI-2.mp4 Two-way nematic-polymer coupling. The polymer is visualized as a black line, the director as white lines and the scalar order parameter S_c as the colour map. The nematic orientation is disturbed around the sudden turn along the polymer. This is indicated by a lower scalar order parameter S_c in the vicinity of the turn. Although all presented quantitative results are in 3D, this simulation is carried out in 2D to visualize the coupling. The polymer consists of $N = 20$ monomers, with a coupling parameter $k = 20$, embedded in a square domain of $L = 30$ with periodic boundary conditions.

Video SI-3.mp4 Hairpin formation. An instance of a single hairpins forming at right-hand end of the polymer can be observed early in the video. This single hairpin is short lived. Additionally, two long-lasting hairpins arise through a pair creation event. Both of these hairpins eventually diffuses to the left-hand end of the polymer. The polymer consists of $N = 20$ monomers, with a coupling parameter $k = 8$, embedded in a perfect-slip cylindrical domain of radius $R = 10$ and length $L = 30$ with periodic boundary conditions. The background nematic with $U = 6$ is transparent to best view the polymer conformation.

Video SI-4.mp4 Hairpin diffusion. Hairpin initially moves diffusively in an anti-clockwise direction along the polymer. The movement then reverses, and the hairpin returns to a position roughly similar to its initial state. The polymer consists of $N = 20$ monomers, with a coupling parameter $k = 10$, embedded in a perfect-slip cylindrical domain of radius $R = 10$ and length $L = 30$ with periodic boundary conditions. The background nematic with $U = 6$ is transparent to best view the polymer conformation.
